# Prevalence and demographics of systemic lupus erythematosus and lupus nephritis among US children with Medicaid coverage, 2002-2004

**DOI:** 10.1186/1546-0096-10-S1-A104

**Published:** 2012-07-13

**Authors:** Linda T Hiraki, Tamara Shaykevich, Wolfgang C Winkelmayer, Karen H Costenbader

**Affiliations:** 1 Brigham and Women's Hospital, Boston, MA, USA; 2 Brigham and Women's Hospital, Harvard School of Public Health, Boston, MA, USA; 3 Department of Medicine, Stanford University School of Medicine, Palo Alto, CA, USA; 4 Harvard School of Public Health, Boston, MA, USA

## Purpose

Little is known about the prevalence or sociodemographics of systemic lupus erythematosus (SLE) among children on Medicaid, the government-funded program which pays for medical care for those who cannot afford it. We investigated nationwide prevalence and sociodemographic characteristics of SLE among children on Medicaid.

## Methods

Children aged 5-19 years with SLE (> 2 ICD-9 codes of 710.0) were identified from Medicaid Analytic eXtract (MAX) data, containing all inpatient and outpatient claims codes for Medicaid patients for all 50 U.S. States, 2002-2004. Within this group, lupus nephritis was identified from billing codes for > 2 of a range of ICD-9 codes for glomerulonephritis, proteinuria and renal failure (PPV 88%, validated by Chibnik et al., 2009). We calculated the prevalence of SLE and lupus nephritis among Medicaid-eligible children and within specific sociodemographic segments.

## Results

Of 25,531,034 children covered by Medicaid from 2002 to 2004, 4515 with SLE were identified: SLE prevalence was 17.7 per 100,000. Of those, 85% were female, 38% Black, 23% Hispanic and 25% White; 43% resided in the South and 23% in the West. Overall, 1655 (37%) of children with SLE had lupus nephritis (prevalence: 6.5 per 100,000). Table 1 shows the prevalence of SLE and lupus nephritis per 100,000 among specific demographic groups of children with Medicaid. Lupus nephritis was more common among Native American (45%), Black (41%), Asian (42%) and Hispanic (35%) children with SLE than among white children (27%).

**Table 1:** Prevalence of SLE and lupus nephritis among US children aged 5-19 years, 2002-2004, (per 100,000 Medicaid-eligible children)

	SLE	Lupus nephritis
All ages 5-19	17.7	6.5
Males	5.5	2.1
Females	29.4	10.7
Blacks	24.6	10.1
Whites	10.4	2.8
Asians	31.4	13.1
Hispanics	20.0	7.0
Native American	28.5	12.9
Northeast	17.2	6.6
South	19.2	7.1
Midwest	14.2	4.8
West	18.6	6.7
Age 5.9	2.7	0.9
Age 10-14	17.7	6.5
Age 15-19	49.5	18.3

## Conclusion

The prevalence of SLE among children with Medicaid medical insurance in the U.S., 2002-2004, was 17.7 per 100,000. The majority was non-white and over a third had been evidence of lupus nephritis. Future studies are required to explore predictors of outcomes of SLE in this population.

## Disclosure

Linda T. Hiraki: None; Tamara Shaykevich: None; Wolfgang C. Winkelmayer: None; Karen H. Costenbader: None.

